# The Impact of Oral Health and Dental Care on Pregnancy: A Cross-Sectional Study Among Women of Reproductive Age

**DOI:** 10.3390/jcm14145153

**Published:** 2025-07-20

**Authors:** Paulina Adamska, Hanna Sobczak-Zagalska, Zuzanna Gromek, Barbara Wojciechowska, Paulina Doroszkiewicz, Marek Chmielewski, Dominika Cichońska, Adam Zedler, Andrea Pilloni

**Affiliations:** 1 Division of Oral Surgery, Medical University of Gdańsk, 7 Dębinki Street, 80-211 Gdańsk, Poland; adam.zedler@gumed.edu.pl; 2 Department of Pediatric Dentistry, Medical University of Gdańsk, 18 Orzeszkowej Street, 80-204 Gdańsk, Poland; h.zagalska@gumed.edu.pl; 3 Scientific Circle of Oral Surgery, Medical University of Gdańsk, 7 Dębinki Street, 80-211 Gdańsk, Poland; zuzannagromek7@gumed.edu.pl (Z.G.); barbara.wojciechowska@gumed.edu.pl (B.W.); p.doroszkiewicz@gumed.edu.pl (P.D.); machmielewski@proton.me (M.C.); 4 Department of Maxillofacial Surgery, Medical University of Gdańsk, 17 Mariana Smoluchowskiego Street, 80-214 Gdańsk, Poland; 5 Private Dental Practice, 14 Kolberga Street, 81-881 Sopot, Poland; 6 Department of Periodontology and Oral Mucosa Diseases, Medical University of Gdańsk, 18 Orzeszkowej Street, 80-204 Gdańsk, Poland; dcichonska@gumed.edu.pl; 7 Section of Periodontics, Department of Oral and Maxillo-Facial Sciences, Sapienza University of Rome, 00-185 Rome, Italy; andrea.pilloni@uniroma1.it

**Keywords:** dental care, dentistry, infant, health services, live birth, low birth weight, oral health, pregnancy, premature birth

## Abstract

**Background**: Prematurely born newborns with low birth weight constitute a group of patients who require special care from the first days of life. Prematurity and low birth weight affect about 13.4 million infants. Risk factors include placental disorders but also factors related to the mother, such as smoking, alcohol drinking, drug use, malnutrition, or certain diseases. It is imperative to educate women of reproductive age (15–49) about the basic factors influencing embryonic development, such as oral health, diet, medicine intake, and harmful habits. Even though most women are aware of the negative impact of harmful habits on the fetus, still too little attention is paid to oral health in pregnant women. Poor oral health may influence the well-being of the future mother, as well as of the child. Therefore, women of reproductive age and those who are pregnant must have adequate knowledge on this subject. The aim of this study was to assess the knowledge of Polish women of reproductive age (15–49) regarding oral health during pregnancy, including the impact of dental treatment, oral hygiene, and maternal oral conditions on pregnancy outcomes and the health of the newborn. **Materials and Methods**: This was a cross-sectional study of 508 women, in the reproductive age, whose age ranged from 18 to 49 years old. The surveys were conducted from April 2020 to November 2020. The questionnaire was originally developed based on the available literature and consisted of seven sections: basic information, general health and habits, pregnancy status and dental care, knowledge of treatment options during pregnancy, oral health status and its association with the risk of preterm birth, prematurity and the child’s oral health, and breastfeeding and oral development. **Results**: After excluding incomplete questionnaires, a total of 499 questionnaires were included in the analysis. Women participating in the study had a fairly good understanding of the impact of oral health on the fetus and the role of breastfeeding in the development of the stomatognathic system (from 50% to 70% correct answers). However, even though most respondents had completed higher education (344/68.94%), their knowledge of oral health, preterm birth, and low birth weight was very limited (including the impact of inflammation on the intrauterine development of the child or bacteria and transfer across the placenta). In these sections, the percentage of correct answers ranged from less than 20% to 50%. When analyzing knowledge by age, education, number of births, and place of residence, the highest levels of knowledge were observed among respondents with higher education, particularly those aged 27–32. **Conclusions**: Respondents had a fairly good understanding of the general impact of oral health during pregnancy and recognition of the importance of breastfeeding for infants. However, their knowledge about the impact of bacteria and inflammation in the mother’s oral cavity on prematurity and low birth weight was limited. Therefore, educating women of reproductive age and pregnant women on this topic is essential, as it may help reduce the adverse consequences of prematurity.

## 1. Introduction

Women’s awareness of the impact of oral health on fetal development is crucial. Therefore, women of reproductive age and pregnant women should have sufficient knowledge on this topic. It seems reasonable that oral health issues, including maternal oral hygiene during the perinatal period, should be introduced during school education. Avoiding dental visits before, during, and after pregnancy, as well as poor oral hygiene and nutritional habits, can be detrimental to the mother’s health and the health and development of her unborn child. It has been proven that poor oral health in pregnant women increases the risk of preterm birth, low birth weight, and neonatal and infant mortality [1,2,3,4,5,6,7]. In 2020, up to 13.4 million infants were born prematurely worldwide, meaning before the 37th week of pregnancy [8,9].

Pregnancy is a life-changing experience for a woman, bringing with it a number of physical and emotional challenges that can significantly impact a woman’s life. Pregnancy poses a significant challenge to the body. It involves intense hormonal changes, weight gain, and increased strain on the circulatory system and internal organs. Conditions such as gestational diabetes and hypertension are more common during pregnancy, which can also affect mental health, increasing the risk of postpartum depression. Hormonal changes in pregnant women make them more susceptible to pathogens and plaque accumulation. This is mainly due to increased levels of estrogen, which reduce gingival cell proliferation; that, in turn, leads to a weakening of the epithelial barrier. Increased progesterone promotes vascular changes such as blood micro-circulation and vascular permeability. It leads to exaggerated inflammatory response to dental plaque, pregnancy-associated gingivitis, and periodontitis. Gingivitis and periodontitis contribute to the secretion of inflammatory mediators such as IL-6, IL-8, and TNF-α that enter the bloodstream during normal daily activities, such as brushing teeth or during dental procedures. These cytokines can play a role in the initiation of uterine contractions and cervical ripening, potentially leading to preterm birth [10,11,12,13]. During pregnancy, increased gastric acid secretion and reflux are observed. This, together with pregnancy-induced vomiting, lead to a decrease in the amount of saliva, its pH, and buffering capacity. The ability of remineralization is disturbed, which favors the occurrence of non-carious lesions, such as enamel erosion [10,11,12,13,14].

During pregnancy, a woman’s dietary intake often changes to meet the increased nutritional demands of both the mother and the developing fetus. These changes may include increased food consumption, adjustments in meal frequency, and greater attention to nutrient-rich foods to support fetal growth, maternal health, and pregnancy outcomes. Moreover, pregnant women have food cravings that are often rich in simple carbohydrates—a substrate for cariogenic bacteria It is very important for women to maintain proper oral hygiene during pregnancy. A good oral health routine should involve teeth brushing twice a day with fluoride paste; tongue cleaning; flossing; and mouth rinsing with alcohol-free, fluoride mouthwashes. Rinsing the mouth with water after vomiting and delaying tooth brushing for approximately 30 min has been suggested in the literature as a method to minimize enamel erosion. Moreover, it is suggested to brush the teeth after every meal. In the second and third trimester, women should use xylitol and alcohol-free chlorhexidine mouthwashes (0.12%) daily [3].

The oral microbiome changes during pregnancy, as pregnant women are more susceptible to microbiota imbalances due to hormonal and immunological shifts. The use of probiotics, paraprobiotics, postbiotics, and minimally invasive disinfection techniques helps regulate oral dysbiosis by reducing pathogenic bacterial complexes. These methods reduce inflammation without using aggressive pharmacological interventions [15]. As in matters of dental treatment, guidelines emphasize that dental treatment can be performed during the whole pregnancy, but the most appropriate time is between 14 and 20 weeks. Some authors suggest that dental treatment during the first trimester may carry some risk, as it is the time of intensive fetal development. That is why they warn to treat those patients only if it is absolutely necessary, and caution is advised [16,17,18,19,20]. Dental appointments and performed procedures in the third trimester should be quick and stress free. The last months of pregnancy are also a good time to educate a mother-to-be about oral hygiene and caries prophylaxis for her infant. Professional dental hygiene and conservative, endodontic, and prosthetic procedures performed during pregnancy typically proceed without significant complications. However, it is recommended to schedule teeth extractions for the second trimester. In emergency cases, treatment should be performed regardless of trimester, prioritizing the mother’s health, the child’s well-being, and the overall pregnancy.

There are numerous medicines that are safe to take during pregnancy, particularly when prescribed appropriately and in the correct dosage. Commonly accepted medications include painkillers, like acetaminophen, antibiotics such as penicillin or amoxicillin, and some antihistamines like loratadine for allergies. However, prescribing nonsteroidal anti-inflammatory drugs (NSAIDs) such as naproxen, ibuprofen, and nimesulide should be avoided due to the risk of miscarriage, premature closure of the ductus arteriosus, and other complications. Local anesthetic solutions such as mepivacaine, bupivacaine, and articaine with adrenaline should be administered with caution [16,17,18,19,20].

The aim of this study was to assess the knowledge of Polish women regarding oral health during pregnancy, including the impact of dental treatment, oral hygiene, and maternal oral conditions on pregnancy outcomes and the health of the newborn.

## 2. Materials and Methods

This was a cross-sectional study of 508 women, in their reproductive age, with ages ranging from 18 to 49 years old. The surveys were conducted from April 2020 to November 2020. The survey was distributed via social media platforms, targeting motherhood-related pages and groups, using a convenience sampling method during the COVID-19 restrictions. All participants provided informed consent to participate in the study. Given the sensitivity of topics such as miscarriage and smoking, participants were informed that their responses were fully anonymous and would be used solely for scientific purposes. Data were encrypted and stored on secure servers. This information was included in the consent form and reiterated prior to data collection. The research was approved by the institutional ethics committee (Independent Bioethics Commission for Research, Medical University of Gdańsk, number of approval NKBBN/443/2019). The study complied with the Declaration of Helsinki and followed the Standards for Reporting of Diagnostic Accuracy guidelines.

### 2.1. Inclusion and Exclusion Criteria

The study included adult women of reproductive age with full rights, literate in reading and writing, and willing to participate in the study by signing an informed consent form, who fully completed the questionnaire.

The study excluded men, minors under the age of 18, women before child-bearing age and postmenopausal, and those who refused to participate in the survey or incapacitated individuals.

### 2.2. Questionnaire

The questionnaire was prepared originally and was based on the available literature [1,2,3,4,5,6,7,10,11,15,18,19,20]. It consisted of seven parts: (I) basic information (age, education, place of residence, marital status, employment, number of pregnancies, and number of live births); (II) general health and habits (chronic diseases, medications, smoking); (III) pregnancy status and dental care; (IV) knowledge about treatment options during pregnancy; (V) knowledge about oral health status and the risk of preterm birth; (VI) knowledge about prematurity and the child’s oral health; and (VII) knowledge about breastfeeding and oral development. Completion of the questionnaire took about 20 min.

In this study, the terms oral health and dental care are used to describe related but distinct aspects: oral health refers to the general condition of the mouth, teeth, and gums, while dental care encompasses professional services and individual hygiene practices aimed at maintaining oral health. For clarity, terminology has been standardized throughout the manuscript. Preterm birth and prematurity are used interchangeably in this manuscript to refer to births occurring before 37 weeks of gestation. The educational levels referenced in this study are based on the Polish education system. Primary education refers to schooling up to approximately age 15, secondary education includes high school or vocational training, and higher education refers to university-level studies.

The questionnaire was pre-tested by women researchers and lecturers of the Medical University of Gdańsk.

The questionnaire was also shared on social media platforms.

### 2.3. Statistical Analysis

The data were analyzed using Statistica v. 13.3 (TIBCO, Palo Alto, CA, USA), licensed to the Medical University of Gdańsk. The sample size was calculated based on the estimated number of medical and dental students, derived from statistical data (population size *N* = 8,500,000, confidence level = 95%, margin of error e = 5%). A minimum of approximately 384 participants was required to obtain representative results.

Normal distribution of the analyzed variables was verified with the Shapiro–Wilk test. The descriptive statistics calculated the number of observations (N), average (M), median (Me), minimum value (Min), maximum value (Max), and standard deviation (SD). In the analysis of relationships between features, the Pearson χ^2^ independence test and Fisher’s exact test were used, depending on expected frequencies. For correlation analysis, Pearson’s r was applied for normally distributed variables, while Spearman’s rho was used for non-normally distributed or ordinal variables. The choice between parametric and nonparametric tests was based on the results of the Shapiro–Wilk test, which was used to assess the normality of distribution for all continuous variables. All tests were considered statistically significant at *p* ≤ 0.05.

## 3. Results

### 3.1. Characteristic of the Study Group

The study involved 508 participants. After excluding incomplete questionnaires, 499 remained and were included in the analysis. The age of the participants was between 19 and 47 years old (mean age 29.49, standard deviation 4.49). Overall, 344 respondents (68.94%) had a higher education, 153 (30.66%) a secondary education, and 2 (0.4%) a primary education. About two thirds of the respondents (70.14%) lived in cities, of which 159 (32%) had more than 300 thousand inhabitants. One hundred and forty-nine (29.86%) participants lived in the countryside. A total of 472 (94.55%) respondents were in relationships (marital or civil partnership), while 27 (5.45%) were single. Three hundred and sixty-two (72.55%) worked full time or were self-employed; the rest managed the households (housewives) or were unemployed (27.45%).

Four hundred and ten (82.16%) respondents were generally healthy. In total, 89 (17.84%) respondents suffered from chronic diseases, but only 82 of them (6.43%) required drug therapy. Among chronic diseases, hypothyroidism was the most common condition among the participants (51/10.22%). One hundred and forty-nine respondents were smokers (32/6.41%) or former smokers (117/23.45%). Of those who smoked, most were light (less than 10 cigarettes a day, 17/3.41%) or moderate (from 10 to less than 20 cigarettes per day, 15/3.01) smokers. The average number of cigarettes smoked was nine (SD = 6), and the number of years was 7.03 (SD = 4.35).

### 3.2. Gynecological and Obstetric Characteristics of the Study Group

More than half of surveyed women were pregnant once (267/53.61%), one-third twice (165/33.13%), and three or more pregnancies were reported by 66 (13.25%) respondents. At the time of completing the questionnaire, one-third of the participants were pregnant (166/33.27%).

The most common pregnancy comorbidities reported by respondents were diabetes (68/13.63%) and hypertension (39/7.82%). The most common pregnancy complaints mentioned by women included nausea (338/52.51%) and vomiting (207/41.48%). Correct oral procedures after vomiting, i.e., rinsing the mouth and brushing the teeth after approximately 30 min, were reported only by 8.22% of the respondents.

Preterm birth (before 37 weeks of pregnancy) occurred in 36 participants (7.35%) and was associated with the low birth weight of the baby (less than 2500 g; 7.35%). Ninety-three (18.64%) women reported a history of miscarriage.

### 3.3. Routine Oral Hygiene and Health of Participants

Appropriate teeth brushing was reported by 424/84.97% respondents (two minutes in the morning and in the evening). The most commonly used toothbrushes were those with medium bristles, both electric (198/39.68%) and manual (156/31.26%). Additional hygiene products mentioned by participants included mouthwash, dental floss, interdental brushes, and irrigators. Table 1 presents detailed information about oral hygiene routine.

Almost half of the respondents stated that they visit the dentist every six months (245/49.2%). One quarter of women reported going to the dental office only once a year (127/25.50%) and 80/16.06% even less than once a year. Moreover, 45/9.24% went to the dentist only as needed and in case of pain. In the six months preceding this survey, 341/68.34% of the participants had visited a dentist.

When asked about the number of meals consumed during the day, respondents most often mentioned five (239/47.99%), then they reported four (117/23.49%). Slightly more respondents indicated more than five (111/22.29%) meals a day, and 31/6.22% only three meals. Hunger pangs occurred in 280 (58.11%) of the participants. Four hundred and seventy (94.19%) of respondents allowed themselves to snack. Additionally, night-time snacking was reported by 87 women (17.43%).

### 3.4. Respondents’ Dental Problems During Pregnancy

Three hundred and twenty (64.14%) of women claimed that they had completed dental treatment before a planned pregnancy. However, 226/46.22% participants underwent dental treatment during pregnancy. The second trimester (105/46.45%) was most often declared as the time when the respondent had completed dental treatment; less frequently, they indicated the first trimester (60/26.55%). Thirty six (15.93%) responders visited the dentist throughout their pregnancy, and 25/11.06%. only in the third trimester. The dental procedures performed included calculus removal (75/33.04%), root canal treatment (25/10.96), and tooth extraction (21/9.25%). Twelve (5.31%) participants underwent radiological examination, 116/52.33% were given anesthesia for treatment, and 9/3.96% received antibiotics.

During pregnancy, as many as 225/45.82% women reported swollen and bleeding gums. Pregnancy epulis was present in nine respondents (1.82%). In addition, 90.16% participants believed that pregnancy had no effect on the oral health of pregnant women.

### 3.5. Knowledge About Dental Care and Treatment During Pregnancy

**Figure 1:**
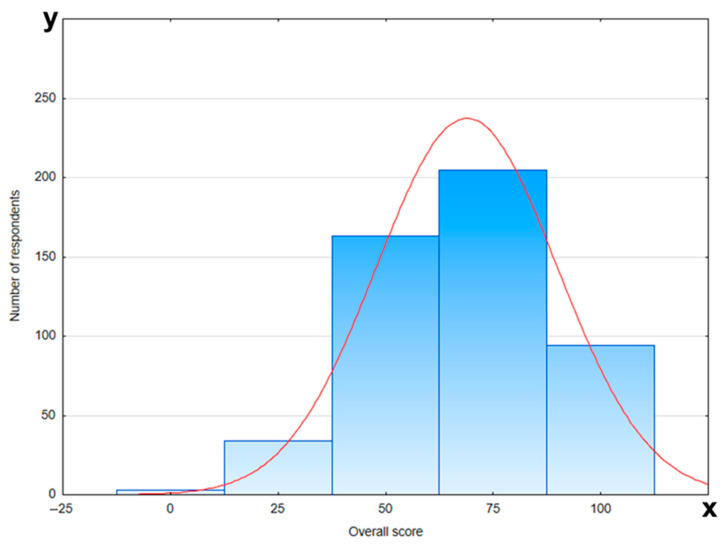
Overall score on knowledge about dental care and treatment during pregnancy (red line—normal distribution (Gaussian curve); x-axis—number of responders; y-axis—overall score).

The level of knowledge of the respondents was assessed in relation to the demographic factors such as age, education, place of living, and number of pregnancies. The group with the greatest knowledge turned out to be respondents aged 27–32 with higher education status. However, the place of living or the number of pregnancies had no influence on the knowledge of the respondents. The respondents gave the worst answers to questions about the possibility of performing tooth extraction and dental X-ray examinations during pregnancy (less than 50% of correct answers). Among women who had only experienced miscarriages, the knowledge about the transmission of dental caries to their child was the lowest (*p* < 0.001). Table 2 presents detailed information about knowledge of dental care and treatment during pregnancy. The graph presenting the overall score on knowledge about dental care and treatment during pregnancy is shown in Figure 1.

Correlation analysis was also performed with the overall result of this part of the study. The individuals with the most correct answers were women over 32 years old (r/R = 0.12, *p* = 0.008), with higher education status (r/R = 0.22, *p* < 0.001), and living in a large city (r/R = 0.18, *p* < 0.001).

### 3.6. Knowledge About Oral Health, Preterm Birth, and Low Birth Weight of the Fetus

**Figure 2:**
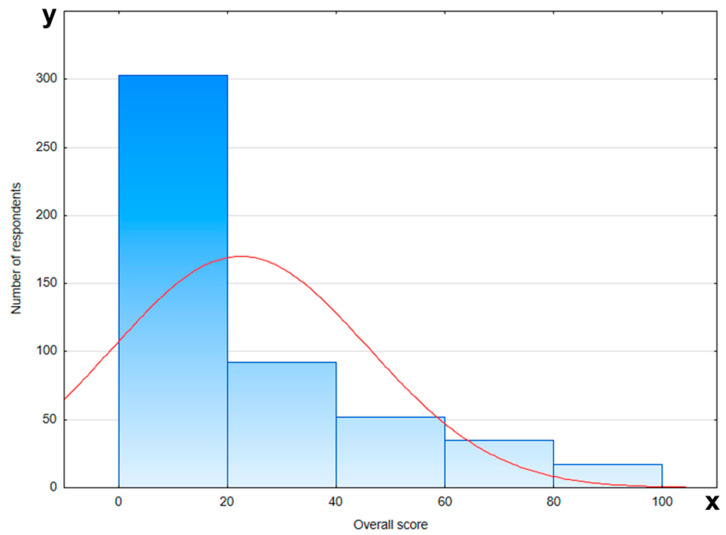
Overall score on knowledge about oral health, preterm birth, and lower birth weight of the fetus (red line—normal distribution (Gaussian curve); x-axis—number of responders; y-axis—overall score).

The level of knowledge of the respondents was assessed in relation to demographic factors such as age, education, place of residence, and number of pregnancies. Analyzing the results, it can be concluded that the respondents did not have sufficient knowledge, because in none of the questions did the level of correct answers exceed 50%. The graph presenting the overall score on knowledge about oral health, preterm birth, and low birth weight of the fetus is shown in Figure 2. Respondents below the age of 26, with primary or secondary education, demonstrated the lowest levels of knowledge. The place of living or the number of pregnancies had no impact on the knowledge of the respondents. Table 3 provides detailed information about participants’ knowledge of oral health, preterm birth, and low birth weight of the fetus.

Correlation analysis was also performed with the overall result of this part of the study. The most correct answers were given by women with higher education (r/R = 0.14, *p* = 0.001) and living in a large city (r/R = 0.11, *p* = 0.014).

### 3.7. Knowledge About the Influence of Prematurity on the Oral Health of the Child

**Figure 3:**
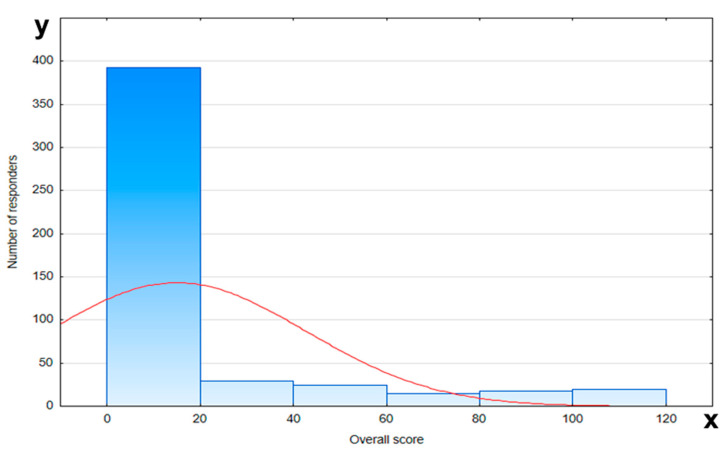
Overall score on knowledge about prematurity and oral health (red line—normal distribution (Gaussian curve); x-axis—number of responders; y-axis—overall score).

The level of respondents’ knowledge was assessed in relation to demographic factors such as age, education, place of residence, and number of pregnancies. Except for two questions, participants did not exceed 20% correct answers in the remaining ones. The overall score on knowledge about prematurity and the oral health of the child is presented in Figure 3. The worst results were achieved by the group of respondents under the age of 26 with primary and secondary education. The number of pregnancies and place of residence had no effect on the level of knowledge. Detailed analysis of the results regarding knowledge about oral health and prematurity is presented in Table 4.

Correlation analysis was also performed with the overall result of this part of the study. The most correct answers were given by women with higher education (r/R = 0.11, *p* = 0.017) and living in a large city (r/R = 0.11, *p* = 0.014).

### 3.8. Knowledge About the Influence of Breastfeeding on the Oral Health of the Child

**Figure 4:**
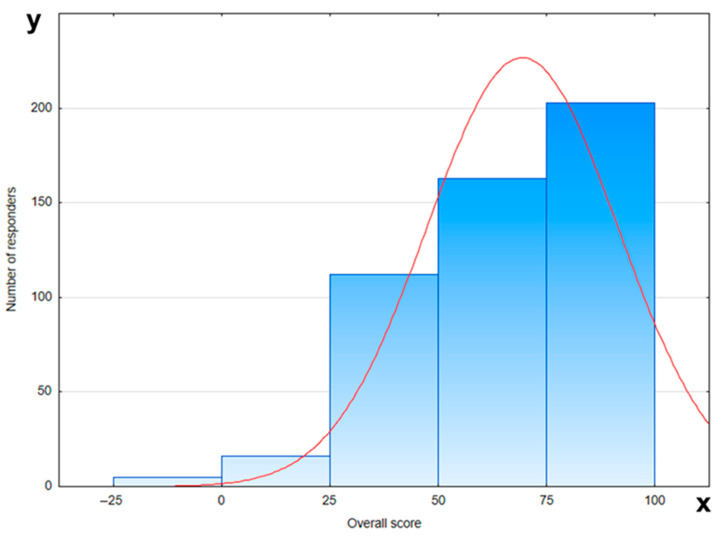
Overall score on knowledge about breastfeeding and oral health of child (red line—normal distribution (Gaussian curve); x-axis—number of responders; y-axis—overall score).

The level of participants’ knowledge was assessed in relation to demographic factors such as age, education, place of residence, and number of pregnancies. It seems that questions regarding this issue were not a problem for the respondents. Less than 30% of women correctly answered the question about the correlation between prolonged breastfeeding and an increased risk of dental caries in children. Figure 4 presents the overall score on knowledge of breastfeeding and the oral health of the child.

Respondents aged between 27 and 33 years and those over 33 years of age with higher education demonstrated the highest level of knowledge. Again, place of residence and number of pregnancies had no influence on knowledge of this subject. Detailed information about knowledge of the relationship between infants’ oral health and breastfeeding is provided in Table 5.

Correlation analysis was also performed with the overall result of this part of the study. The most correct answers were given by women from the oldest age group (r/R = 0.15, *p* = 0.001), with higher education (r/R = 0.22, *p* < 0.001), and living in a large city (r/R = 0.15, *p* < 0.001).

## 4. Discussion

Parents’ knowledge and oral health awareness are among the factors influencing the development and well-being of their children. It is essential that individuals, both women and men, planning the pregnancy possess the understanding that oral health is a part of prenatal care. Poor oral health during pregnancy can lead to negative health outcomes for the mother and the child. The oral cavity is colonized by many different bacteria species that can enter the circulatory system even during eating or tooth brushing. Such presence of viable bacteria in the circulating blood is called bacteriemia. Failure to eliminate oral inflammation before pregnancy may have adverse effects on pregnancy outcomes, the child’s birth weight, and may even lead to preterm delivery or fetal death [1,2,3,4,5,6,7,8,9].

Most study participants followed common recommendations on tooth brushing. Almost 85% of respondents brushed their teeth twice a day for 2 min using the appropriate hardness of the toothbrush bristles (medium or soft) [21,22]. Unfortunately, flossing was reported only by about 40% of women. One third used mouthwashes. These results are disturbing, as flossing and mouth rinsing significantly reduce the amount of plaque and lower the risk of gingivitis or periodontitis [23,24,25]. Less than 10% of respondents used an interdental toothbrush or irrigator. Nearly half of the participants regularly visited the dentist. However, what is alarming is that almost 36% of questioned women did not treat any oral health problems in advance of planned pregnancy. Another disturbing issue was snacking between meals, which was reported by almost 95% of the respondents. This can contribute to tooth decay [26,27,28].

Almost half of the participants of our study had to undergo dental treatment during pregnancy. This could be a consequence of the lack of a dental full check-up before pregnancy. Women were mainly treated in the second trimester, which is recommended because it is the safest time to perform dental procedures [16,17,18,19,20,29,30]. The respondents were aware that the second trimester is the best time for treatment (over 97%). However, the knowledge that dental treatment, if necessary, can be performed at any time during pregnancy was lower (approx. 70%). This may be due to the belief that dental procedures are not safe for pregnant women. It is much more important to eliminate inflammation and carious lesions in the oral cavity of a pregnant woman than to expose her and the fetus to bacteria and the stress associated with pain related to untreated caries. That is why dental treatment can be performed throughout pregnancy [11,30,31]. This is particularly important because, during pregnancy, the risk of caries and periodontal disease increases, and periodontal parameters deteriorate [11,30]. About 70% of respondents were aware of these threats. In addition, some bacteria can be transmitted from mother to child, e.g., Streptococcus mutans infection. Hence, the lower the level of Streptococcus mutans in the mother’s oral cavity, which is associated, among other things, with the lack of carious lesions, the lower the number of these bacteria in the child [32].

The dental procedures most frequently performed by pregnant participants of our study were mainly associated with professional oral hygiene (scaling, root planing, and polishing), and almost three-quarters of women found these procedures to be safe during pregnancy. This is justified because professional dental hygiene reduces pathological pathogens in the gingival pockets [20,33,34]. More than 90% of respondents knew that dental caries can be treated during pregnancy, but only 60% considered endodontic treatment to be possible to perform in pregnant women. According to the studies, both caries and root canal treatment are indicated during the gestational period whenever necessary [31,35]. Radiation doses from dental X-rays are low, and if diagnosis for treatment is necessary, dental X-rays must be performed [36]. It is similar with anesthesia, because increased stress during the procedure affects, among other things, the increase in blood pressure and can lead to greater complications than anesthesia itself [31,35]. Our study revealed that one third of women were aware that dental X-rays can be performed during pregnancy, but as many as three quarters knew about the possibility of using anesthesia in the expectant mother. In exceptional situations, if it is a health risk, teeth extractions are recommended, and antibiotics, which are safe for the fetus, may be administered [31,34]. Less than 30% of study participants considered extractions as possible to perform during pregnancy, while more than 80% claimed antibiotics could be taken.

Interestingly, 90% of women believed that pregnancy did not affect their oral health. In the study group, almost 50% of respondents experienced bleeding and swelling of their gums during pregnancy, and less than 2% experienced pregnancy epulis, which is typical for pregnant women. According to studies, the prevalence of gingivitis is estimated at 30–100% [37,38] and pregnancy epulis at 5% [39].

More than half of the respondents suffered from nausea or vomiting during pregnancy. However, less than 10% knew how to act properly after vomiting, i.e., immediate rinsing of the mouth and brushing teeth at least 30 min after [40].

The study also analyzed the knowledge of respondents about the connection between oral health and poor pregnancy outcomes such as preterm delivery and infants’ low birth weight. One of the questions concerned the possibility of bacteria reaching the placenta. Only one third of women were aware of such a possibility. It has been proven that bacteria from the oral cavity cross the placenta [33]. These are *Fusobacterium nucleatum*, *Aggregatibacter actinomycetemcomitans*, *Treponema denticola*, *Tannerella forsythia*, *Fusobacterium nucleatum*, and *Campylobacter rectus* [4,5]. These bacteria can also cause intrauterine infection [4,5,6], which only 1/3 of surveyed women knew. Inflammation in the oral cavity is among the factors of premature birth or low birth weight. Dental caries, gangrene of the dental pulp, periodontal diseases, or impaired eruption of wisdom teeth are potential infection foci in the oral cavity and can negatively affect pregnancy [2,5,6,41,42,43]. In these detailed questions, the subjects responded poorly. Correct answers were at the level of 3–35%.

Another issue mentioned in the study was the impact of premature birth on the baby’s stomatognathic system. It is assumed that preterm delivery may have deleterious effects on oral health and development. Increased incidence of gingivitis, lower salivary secretion [44], higher number of cariogenic bacteria [45], enamel hypomineralization of primary and permanent teeth [46], and early childhood caries [47,48] are some of the oral effects of preterm birth. The questions regarding prematurity and its impact on postnatal oral development were very difficult for our respondents, as only one in ten of them gave correct answers.

Breastfeeding is crucial for the proper and harmonious development of the stomatognathic system [49]. In cases where the mother is unable to breastfeed (low milk supply, breast inflammation, or illness), bottle-feeding with formula milk is used as an alternative. It is essential to use proper positioning during bottle-feeding to allow free protrusion of the mandible and not to hinder its development [50]. Bottle-feeding, especially at night, increases the risk of tooth decay in the child. In the case of nighttime breastfeeding, this is a controversial issue. Some researchers believe that there is no such connection [51]. A detailed analysis of publications by Branger’s team [52] found that up to the age of one, breastfeeding protects against caries, in contrast to formula feeding. However, after the first year of life, the situation changes, and breastfeeding may be a risk factor of early childhood caries. Branger et al. [52] point out that studies are not consistent, as they often lack an analysis of nighttime feeding, the number of meals or snacks, and oral hygiene. Expanding a baby’s diet with solid foods can start around 6 months of age [53]. The respondents performed quite well in this area, and a large percentage had adequate knowledge about breastfeeding. The most challenging question concerned extended breastfeeding. The WHO recommends continuing breastfeeding for as long as the mother or child need. However, without proper oral hygiene and diet habits, extended breastfeeding can sometimes lead to teeth caries.

Barbosa et al. presented a good knowledge of pregnant women about the importance of oral health; however, the risks of dental treatment during pregnancy were commonly observed. Therefore, the need for guidance on oral health in pregnancy is essential [54].

Another questionnaire study conducted in Poland concluded that the overall oral health attitude among pregnant women was modified by sociodemographic factors. However, the most important factors were the level of knowledge and the use of dental care before pregnancy rather than level of education and urban residence. Attending dental visits was related to the conviction about their safety, which emphasized the importance of a proper education of future mothers in this field [55]. Radwan-Oczko et al. [56] observed a proper knowledge of the importance of oral health during pregnancy, which was correlated with higher education status and urban residence. Therefore, a wider education in this field should be provided [56].

However, in a questionnaire study conducted in Italy, more than 40% of women were not aware of a correlation between oral health and pregnancy, and almost three quarters of participants had not received any advice about their oral health during pregnancy. Higher knowledge was associated with the number of children and receiving information about oral health during pregnancy [57].

Petit et al. [58], in a questionnaire study conducted on women between 1 and 3 days after delivery in France, reported high knowledge of the importance of oral diseases prevention during pregnancy; however, only a few of them discussed oral health consideration with a health professional [58].

Low birth weight and prematurity not only affect the development of the facial skeleton and oral cavity (as discussed in this paper) but also have significant implications for overall health. In Poland, the incidence of low birth weight is approximately 6%, while premature births occur in 6–7% of cases. The most common complications include infections (such as pneumonia, sepsis, and necrotizing enterocolitis); retinopathy; delayed motor and/or intellectual development; and, in the long term, difficulties with concentration, reading, and speaking. This underscores the importance of preventive measures and efforts to mitigate adverse health outcomes, as the consequences can persist throughout a child’s life.

The study’s findings are concerning, especially considering that basic dental care for pregnant women is free of charge in Poland. It includes dental checkups, conservative treatment, endodontic procedures, periodontal treatment (such as scaling, root planing, and curettage), and surgical procedures (excluding implantology). The pregnancy card/book, which every pregnant woman in Poland is required to have, includes a space for recording mandatory dental visits and any dental treatment. Unfortunately, the lack of recording women’s dental visits in their pregnancy cards has no legal consequences and is not monitored by the relevant authorities. This issue clearly requires change. A more rigorous approach is necessary, for example, by imposing consequences for the lack of regular dental visits during pregnancy. Such consequences could include losing the woman’s right to future subsidized dental care, having to pay the full cost of dental treatment, and losing access to certain government reimbursements. The study has several limitations. Firstly, the study group was heterogeneous in terms of demographic and socio-economic background. Additionally, the participants represented a wide age range, which implies that they were exposed to different standards of school-based and prenatal education. This variation may have influenced their knowledge and awareness of oral health during pregnancy. Secondly, the study relied on self-reported data, which may be subject to recall bias or social desirability bias. Thirdly, the survey was conducted in a limited geographical area, which may reduce the generalizability of the results to the entire population of Polish women.

Despite its limitations, this study offers novel insights by exploring the awareness and attitudes toward oral health during pregnancy in a real-world population of Polish women with diverse demographic and socio-economic backgrounds. Unlike previous studies, this article reflects the everyday experiences and educational variability of a broader segment of society. Additionally, by collecting self-reported data, the study captures subjective perceptions and personal health behaviors, which are crucial for designing effective public health interventions and educational programs. The research also contributes region-specific data that can inform future national policies on maternal oral health.

Future research should focus on natural substances such as paraprobiotics to avoid dysbiosis during pregnancy that can cause damage to the fetus and the pregnant woman [59].

Moreover, based on the authors’ experience as educators in childbirth preparation classes, the topic of dental care and the impact of oral inflammatory conditions on fetal health are not routinely addressed in Poland. As a result, many parents lack sufficient knowledge in this area. Therefore, new and intensified oral health promotion strategies should be developed and implemented, particularly within prenatal education frameworks.

## 5. Conclusions

The respondents demonstrated a quite good understanding of dental care during pregnancy and recognized the importance of breastfeeding for infants. However, their knowledge of the impact of bacteria and inflammation in the mother’s oral cavity on prematurity and low birth weight was limited. Therefore, educational programs addressing the importance of oral health before, during, and after pregnancy are essential for future parents. What is more, it seems justified to also include in such education people from the closest environment. Many studies have focused on assessing women’s knowledge about oral health and its implications for the course of pregnancy and the development and health of the child. However, the number of studies involving men and people close to pregnant women appears to be much smaller. The adage ‘prevention is better than cure’ is universal and timeless. Regular dental care for women can reduce the risk of premature delivery and the adverse consequences of prematurity.

Proactive measures should also be implemented, such as incorporating oral health education into prenatal care programs and conducting social campaigns targeted at couples planning a pregnancy. Dental and gynecological/obstetrics lines should be involved in programs promoting oral health during pregnancy and preventive dental screenings for women planning a pregnancy. Furthermore, it is essential to educate healthcare professionals—especially obstetricians and midwives—on the importance of oral health. These activities can help translate knowledge into preventive practices aimed at protecting both the mother and the developing child.

## Figures and Tables

**Table 1:** Oral hygiene routine in study group.

	Mouth Rinse		Tooth Floss		Interdental Toothbrush		Irrigator	
	N	%	N	%	N	%	N	%
Yes	145	29.06	202	40.48	33	6.61	28	5.61
No	354	70.94	297	59.52	466	93.39	471	94.39

**Table 2:** Knowledge about dental care and treatment during pregnancy (* *p =* 0.05).

**Pregnancy and Dental Treatment**	**Age Group**				**Statistics**				**Place of Living (Thousands)**					**Statistics**			
	**<26 y**	**27–33 y**	**>33 y**	**General**	**Chi**	**df**	***p***	**Fi**	**Countryside**	**<100**	**100–300**	**>300**	**General**	**Chi**	**df**	***p***	**Fi**
Can dental treatment be performed during pregnancy?	93.33%	98.85%	97.46%	97.19%	9.22	2	* 0.010	0.14	95.30%	96.67%	100.00%	98.11%	97.19%	4.62	3	0.202	0.10
Can dental treatment be performed throughout pregnancy?	60.00%	77.39%	75.42%	72.72%	13.10	2	* 0.001	0.16	71.81%	62.50%	81.69%	77.36%	72.75%	10.99	3	* 0.012	0.15
What is the best time for dental treatment during pregnancy?	50.00%	64.75%	54.24%	58.72%	8.66	2	* 0.013	0.13	48.99%	62.50%	63.38%	62.89%	58.72%	8.30	3	* 0.040	0.13
Can calculus be removed during pregnancy?	66.67%	73.95%	77.97%	73.15%	4.05	2	0.132	0.09	63.76%	75.00%	70.42%	81.76%	73.15%	13.17	3	* 0.004	0.16
Can cavities be treated during pregnancy?	85.00%	92.34%	94.92%	91.18%	8.18	2	* 0.017	0.13	88.59%	86.67%	97.18%	94.34%	91.18%	9.44	3	* 0.024	0.14
Can root canal treatment be performed during pregnancy?	50.00%	63.22%	61.86%	59.72%	6.27	2	* 0.044	0.11	47.65%	61.67%	59.15%	69.81%	59.72%	15.95	3	* 0.001	0.18
Can dental radiographic examinations be performed during pregnancy?	25.00%	35.25%	33.05%	32.26%	3.99	2	0.136	0.09	19.46%	37.50%	36.62%	38.36%	32.26%	16.00	3	* 0.001	0.18
Can local anesthesia be used during pregnancy?	70.00%	78.93%	76.27%	76.15%	3.61	2	0.165	0.09	66.44%	78.33%	80.28%	81.76%	76.15%	11.47	3	* 0.009	0.15
Can tooth extractions be performed during pregnancy?	21.67%	32.95%	25.42%	28.46%	5.84%	2	0.054	0.11	26.85%	29.17%	25.35%	30.82%	28.46%	0.99	3	0.803	0.04
Can antibiotics be administered during pregnancy?	80.00%	81.99%	91.53%	83.77%	7.08	2	* 0.029	0.12	81.88%	85.00%	80.28%	86.16%	83.77%	1.83	3	0.608	0.06
Does the risk of developing cavities increase during pregnancy?	73.33%	73.18%	66.10%	71.54%	2.25	2	0.325	0.07	71.81%	70.83%	71.83%	71.70%	71.54%	0.04	3	0.998	0.01
Does the risk of developing periodontal disease increase during pregnancy?	68.33%	68.97%	68.64%	68.74%	0.02	2	0.992	0.01	65.77%	71.67%	70.42%	68.55%	68.74%	1.19	3	0.757	0.05
Can a child get dental caries from a parent?	68.33%	84.67%	89.83%	81.96%	21.32	2	* 0.000	0.21	73.83%	80.83%	85.92%	88.68%	81.96%	12.38	3	* 0.006	0.16
**Pregnancy and Dental Treatment**		**Education**			**Statistics**				**Pregnancies**				**Statistics**				
		**Primary and Secondary**	**Tertiary**	**General**	**Chi**	**df**	***p***	**Fi**	**1**	**2**	**3+**	**General**	**Chi**	**df**	***p***		**Fi**
Can dental treatment be performed during pregnancy?		94.29%	98.55%	97.31%	6.91	1	* 0.009	0.12	98.13%	96.97%	93.94%	97.19%	3.44	2	0.179		0.08
Can dental treatment be performed throughout pregnancy?		64.29%	76.45%	72.93%	7.46	1	* 0.006	0.12	67.79%	81.82%	71.21%	72.89%	10.26	2	* 0.006		0.14
What is the best time for dental treatment during pregnancy?		51.43%	62.21%	59.09%	4.78	1	* 0.029	0.12	60.30%	57.58%	54.55%	58.63%	0.84	2	0.658		0.04
Can calculus be removed during pregnancy?		65.71%	76.74%	32.02%	6.22	1	* 0.013	0.11	72.28%	75.76%	69.70%	73.09%	1.07	2	0.585		0.05
Can cavities be treated during pregnancy?		84.29%	94.19%	75.83%	12.31	1	* 0.000	0.16	89.51%	92.12%	95.45%	91.16%	2.60	2	0.273		0.07
Can root canal treatment be performed during pregnancy?		51.43%	63.37%	28.31%	5.91	1	* 0.015	0.11	56.93%	65.45%	56.06%	59.64%	3.48	2	0.175		0.08
Can dental radiographic examinations be performed during pregnancy?		22.14%	36.05%	32.03%	8.84	1	* 0.003	0.14	26.97%	38.18%	37.88%	32.13%	7.04	2	* 0.030		0.12
Can local anesthesia be used during pregnancy?		69.29%	78.49%	75.83%	4.60	1	* 0.032	0.10	74.16%	79.39%	75.76%	76.10%	1.54	2	0.462		0.06
Can tooth extractions be performed during pregnancy?		17.86%	32.56%	28.31%	10.60	1	* 0.001	0.15	27.72%	33.33%	19.70%	28.51%	4.48	2	0.106		0.09
Can antibiotics be administered during pregnancy?		75.00%	87.79%	84.09%	12.17	1	* 0.000	0.16	82.02%	86.67%	83.33%	83.73%	1.62	2	0.444		0.06
Does the risk of developing cavities increase during pregnancy?		65.71%	73.55%	71.28%	2.98	1	0.084	0.08	74.91%	67.27%	68.18%	71.49%	3.32	2	0.190		0.08
Does the risk of developing periodontal disease increase during pregnancy?		65.00%	70.64%	69.01%	1.48	1	0.224	0.06	72.28%	66.06%	60.61%	68.67%	4.14	2	0.126		0.09
Can a child get dental caries from a parent?		70.71%	87.21%	82.44%	18.70	1	* 0.000	0.20	77.53%	89.09%	83.33%	82.13%	9.36	2	* 0.009		0.14

**Table 3:** Knowledge about oral health and preterm birth factors (* *p =* 0.05).

**Oral Health and Preterm Birth Factors**	**Age Group**				**Statistics**				**Place of Living (Thousands)**					**Statistics**			
	**<26 y**	**27–33 y**	**>33 y**	**General**	**Chi**	**df**	***p***	**Fi**	**Countryside**	**<100**	**100–300**	**>300**	**General**	**Chi**	**df**	***p***	**Fi**
Can bacteria from the mother’s mouth cross the blood–placenta barrier?	29.17%	34.10%	30.51%	32.06%	1.09	2	0.580	0.05	28.86%	33.33%	36.62%	32.08%	32.06%	1.47	3	0.690	0.05
Can bacteria in the mother’s mouth cause an intrauterine infection?	25.83%	38.70%	33.90%	34.47%	6.04	2	0.049	0.11	28.86%	36.67%	39.44%	35.85%	34.47%	3.24	3	0.356	0.08
Can maternal dental caries contribute to preterm birth?	40.83%	46.74%	50.85%	46.29%	2.44	2	0.295	0.07	36.91%	49.17%	49.30%	51.57%	46.29%	7.71	3	0.052	0.12
Can maternal tooth gangrene contribute to preterm birth?	21.67%	34.87%	32.20%	31.06%	6.78	2	* 0.034	0.12	21.48%	32.50%	32.39%	38.36%	31.06%	10.53	3	* 0.015	0.15
Can maternal periodontal disease contribute to preterm birth?	23.33%	34.10%	26.27%	29.66%	5.42	2	0.067	0.10	20.81%	31.67%	30.99%	35.85%	29.66%	8.81	3	* 0.032	0.13
Can factors released into the bloodstream during maternal periodontal disease contribute to preterm birth?	25.83%	36.02%	34.75%	33.27%	3.99	2	0.136	0.09	25.50%	38.33%	28.17%	38.99%	33.27%	8.61	3	* 0.035	0.13
Can the difficult eruption of the mother’s wisdom teeth contribute to preterm birth?	4.17%	6.13%	3.39%	5.01%	1.52	2	0.468	0.06	3.36%	4.17%	7.04%	6.29%	5.01%	2.20	3	0.532	0.07
Can maternal oral bacteria contribute to preterm birth?	20.83%	30.27%	22.03%	26.05%	5.09	2	0.078	0.10	20.13%	28.33%	29.58%	28.30%	26.05%	3.91	3	0.272	0.09
Can bacteria in the mother’s mouth contribute to the premature rupture of membranes?	5.83%	16.86%	14.41%	13.63%	8.57	2	* 0.014	0.13	9.40%	12.50%	12.68%	18.87%	13.63%	6.16	3	0.104	0.11
Can maternal dental caries lead to a lower birth weight in the fetus?	20.00%	17.62%	12.71%	17.03%	2.37	2	0.306	0.07	18.79%	21.67%	14.08%	13.21%	17.03%	4.23	3	0.237	0.09
Can maternal dental gangrene lead to a lower birth weight in the fetus?	5.83%	16.86%	18.64%	14.63%	10.00	2	* 0.007	0.14	10.74%	12.50%	16.90%	18.87%	14.63%	4.82	3	0.185	0.10
Can periodontal disease in the mother lead to a lower birth weight in the fetus?	6.67%	18.01%	19.49%	15.63%	9.76	2	* 0.008	0.14	13.42%	15.83%	15.49%	17.61%	15.63%	1.03	3	0.795	0.05
Can factors released into the bloodstream during maternal periodontal disease contribute to a lower fetal birth weight?	8.33%	21.07%	17.80%	17.23%	9.39	2	* 0.009	0.14	13.42%	20.00%	16.90%	18.87%	17.23%	2.46	3	0.482	0.07
Can the difficult eruption of wisdom teeth in the mother result in a lower birth weight?	26.67%	25.67%	24.58%	25.65%	0.14	2	0.934	0.02	29.53%	20.00%	25.35%	26.42%	25.65%	3.24	3	0.357	0.08
Can bacteria in the mother’s mouth lead to a lower birth weight in the fetus?	7.50%	19.54%	18.64%	16.43%	9.23	2	* 0.010	0.14	13.42%	15.83%	16.90%	19.50%	16.43%	2.11	3	0.549	0.07
**Oral Health and Preterm Birth Factors**		**Education**			**Statistics**				**Pregnancies**				**Statistics**				
		**Primary and Secondary**	**Tertiary**	**General**	**Chi**	**df**	***p***	**Fi**	**1**	**2**	**3+**	**General**	**Chi**	**df**	***p***		**Fi**
Can bacteria from the mother’s mouth cross the blood–placenta barrier?		29.29%	33.43%	32.23%	0.78	1	0.376	0.04	29.21%	36.36%	31.82%	31.93%	2.40	2	0.301		0.07
Can bacteria in the mother’s mouth cause an intrauterine infection?		30.00%	37.21%	35.12%	2.27	1	0.132	0.07	34.83%	36.97%	27.27%	34.54%	1.98	2	0.371		0.06
Can maternal dental caries contribute to preterm birth?		37.86%	50.29%	46.69%	6.18	1	* 0.013	0.11	46.44%	49.70%	36.36%	46.18%	3.39	2	0.184		0.08
Can maternal tooth gangrene contribute to preterm birth?		20.00%	36.05%	31.40%	11.89	1	* 0.001	0.16	32.21%	32.73%	21.21%	30.92%	3.37	2	0.185		0.08
Can maternal periodontal disease contribute to preterm birth?		22.14%	33.43%	30.17%	6.02	1	* 0.014	0.11	31.46%	30.30%	21.21%	29.72%	2.70	2	0.259		0.07
Can factors released into the bloodstream during maternal periodontal disease contribute to preterm birth?		24.29%	37.79%	33.88%	8.10	1	* 0.004	0.13	34.08%	34.55%	25.76%	33.13%	1.88	2	0.391		0.06
Can the difficult eruption of the mother’s wisdom teeth contribute to preterm birth?		4.29%	5.52%	5.17%	0.31	1	0.577	0.03	6.37%	2.42%	6.06%	5.02%	3.50	2	0.174		0.08
Can maternal oral bacteria contribute to preterm birth?		20.71%	28.78%	26.45%	3.33	1	0.068	0.08	25.47%	27.27%	24.24%	25.90%	0.28	2	0.868		0.02
Can bacteria in the mother’s mouth contribute to the premature rupture of membranes?		10.00%	15.70%	14.05%	2.68	1	0.102	0.07	13.48%	15.76%	9.09%	13.65%	1.79	2	0.408		0.06
Can maternal dental caries lead to a lower birth weight in the fetus?		22.86%	14.83%	17.15%	4.52	1	* 0.034	−0.10	15.73%	17.58%	21.21%	17.07%	1.17	2	0.558		0.05
Can maternal dental gangrene lead to a lower birth weight in the fetus?		8.57%	17.44%	14.88%	6.18	1	* 0.013	0.11	13.11%	17.58%	13.64%	14.66%	1.69	2	0.429		0.06
Can periodontal disease in the mother lead to a lower birth weight in the fetus?		12.14%	17.44%	15.91%	2.09	1	0.148	0.07	14.23%	17.58%	16.67%	15.66%	0.92	2	0.631		0.04
Can factors released into the bloodstream during maternal periodontal disease contribute to a lower fetal birth weight?		10.00%	20.64%	17.56%	7.78	1	* 0.005	0.13	15.73%	21.21%	13.64%	17.27%	2,85	2	0.241		0.08
Can the difficult eruption of wisdom teeth in the mother result in a lower birth weight?		31.43%	23.84%	26.03%	2.98	1	0.084	−0.08	24.34%	24.85%	33.33%	25.70%	2,33	2	0.311		0.07
Can bacteria in the mother’s mouth lead to a lower birth weight in the fetus?		11.43%	18.90%	16.74%	3.98	1	* 0.046	0.09	14.98%	18.79%	16.67%	16.47%	1,08	2	0.584		0.05

**Table 4:** Knowledge about oral health and prematurity (* *p =* 0.05).

**Prematurity and Oral Health**	**Age Group**				**Statistics**				**Place of Living (Thousands)**					**Statistics**			
	**<26 y**	**27–33 y**	**>33 y**	**General**	**Chi**	**df**	***p***	**Fi**	**Countryside**	**<100**	**100–300**	**>300**	**General**	**Chi**	**df**	***p***	**Fi**
Are premature children more prone to gingivitis?	7.50%	12.64%	10.17%	10.82%	2.32	2	0.313	0.07	10.74%	9.17%	11.27%	11.95%	10.82%	0.57	3	0.904	0.03
Do premature babies have reduced salivary flow more often than full-term babies?	14.17%	21.46%	12.71%	17.64%	5.59	2	0.061	0.11	14.09%	20.00%	18.31%	18.87%	17.64%	1.94	3	0.586	0.06
Do premature babies have a higher number of cariogenic bacteria than full-term babies?	10.00%	12.64%	6.78%	10.62%	3.01	2	0.222	0.08	10.74%	10.00%	14.08%	9.43%	10.62%	1.18	3	0.757	0.05
Are premature babies more likely to have enamel hypomineralization than full-term babies?	13.33%	18.77%	15.25%	16.63%	1.97	2	0.374	0.06	14.09%	14.17%	16.90%	20.75%	16.63%	3.17	3	0.366	0.08
Are premature babies more likely to have impaired mineralization of primary teeth than full-term babies?	15.00%	23.37%	17.80%	20.04%	4.08	2	0.130	0.09	18.12%	15.83%	21.13%	24.53%	20.04%	3.72	3	0.293	0.09
Do premature babies experience more severe caries in primary teeth than full-term babies?	10.83%	17.24%	11.02%	14.23%	4.07	2	0.131	0.09	12.75%	12.50%	16.90%	15.72%	14.23%	1.27	3	0.737	0.05
**Prematurity and Oral Health**		**Education**			**Statistics**				**Pregnancies**				**Statistics**				
		**Primary and Secondary**	**Tertiary**	**General**	**Chi**	**df**	***p***	**Fi**	**1**	**2**	**3+**	**General**	**Chi**	**df**	***p***		**Fi**
Are premature children more prone to gingivitis?		7.14%	12.50%	10.95%	2.93	1	0.087	3	11.99%	9.09%	10.61%	10.84%	0.89	2	0.641		0.04
Do premature babies have reduced salivary flow more often than full-term babies?		12.14%	19.77%	17.56%	4.00	1	* 0.046	3	17.23%	20.00%	13.64%	17.67%	1.39	2	0.499		0.05
Do premature babies have a higher number of cariogenic bacteria than full-term babies?		6.43%	11.92%	10.33%	3.24	1	0.072	3	10.11%	11.52%	10.61%	10.64%	0.21	2	0.900		0.02
Are premature babies more likely to have enamel hypomineralization than full-term babies?		12.86%	18.02%	16.53%	1.92	1	0.165	3	14.23%	21.21%	15.15%	16.67%	3.70	2	0.157		0.09
Are premature babies more likely to have impaired mineralization of primary teeth than full-term babies?		14.29%	22.67%	20.25%	4.34	1	* 0.037	3	18.35%	23.03%	19.70%	20.08%	1.40	2	0.497		0.05
Do premature babies experience more severe caries in primary teeth than full-term babies?		8.57%	16.28%	14.05%	4.90	1	* 0.027	3	12.73%	15.76%	16.67%	14.26%	1.12	2	0.570		0.05

**Table 5:** Knowledge about oral health and breastfeeding (* *p =* 0.05).

**Oral Health and Breastfeeding**	**Age Group**				**Statistics**				**Place of Living (Thousands)**					**Statistics**			
	**<26 y**	**27–33 y**	**>33 y**	**General**	**Chi**	**df**	***p***	**Fi**	**Countryside**	**<100**	**100–300**	**>300**	**General**	**Chi**	**df**	***p***	**Fi**
Does breastfeeding affect the development of a child’s chewing system (stomatognathic system)?	82.50%	89.27%	90.68%	87.98%	4.63	2	0.099	0.10	85.91%	85.83%	87.32%	91.82%	87.98%	3.38	3	0.337	0.08
Does bottle-feeding affect the development of the mandible?	80.83%	80.84%	79.66%	80.56%	0.08	2	0.961	0.01	77.18%	74.17%	88.73%	84.91%	80.56%	9.16	3	* 0.027	0.14
Does prolonged breastfeeding increase the risk of tooth decay?	22.50%	27.20%	27.97%	26.25%	1.17	2	0.556	0.05	20.81%	25.83%	23.94%	32.70%	26.25%	5.91	3	0.116	0.11
Does nighttime breastfeeding increase the risk of tooth decay in a child?	35.00%	49.43%	47.46%	45.49%	7.14	2	* 0.028	0.12	40.27%	45.00%	53.52%	47.17%	45.49%	3.68	3	0.298	0.09
Does the type of liquid given to a child affect the risk of tooth decay?	85.00%	94.25%	94.92%	92.18%	11.37	2	* 0.003	0.15	89.93%	90.83%	90.14%	96.23%	92.18%	5.37	3	0.147	0.10
When should a child start eating foods other than breast milk?	78.33%	83.52%	90.68%	83.97%	6.81	2	* 0.033	0.12	79.19%	82.50%	85.92%	88.68%	83.97%	5.54	3	0.137	0.11
**Oral Health and Breastfeeding**		**Education**			**Statistics**				**Pregnancies**				**Statistics**				
		**Primary and Secondary**	**Tertiary**	**General**	**Chi**	**df**	***p***	**Fi**	**1**	**2**	**3+**	**General**	**Chi**	**df**	***p***		**Fi**
Does breastfeeding affect the development of a child’s chewing system (stomatognathic system)?		83.57%	90.99%	88.84%	5.52	1	0.019	0.11	86.89%	90.30%	86.36%	87.95%	1.30	2	0.522		0.05
Does bottle-feeding affect the development of the mandible?		78.57%	82.27%	81.20%	0.89	1	0.345	0.04	79.40%	81.82%	81.82%	80.52%	0.46	2	0.794		0.03
Does prolonged breastfeeding increase the risk of tooth decay?		22.14%	27.91%	26.24%	1.71	1	0.191	0.06	27.72%	24.24%	25.76%	26.31%	0.65	2	0.724		0.04
Does nighttime breastfeeding increase the risk of tooth decay in a child?		37.86%	49.13%	45.87%	5.09	1	0.024	0.10	41.57%	52.12%	45.45%	45.58%	4.57	2	0.102		0.10
Does the type of liquid given to a child affect the risk of tooth decay?		87.86%	94.77%	92.77%	7.08	1	0.008	0.12	90.26%	95.15%	93.94%	92.37%	3.72	2	0.155		0.09
When should a child start eating foods other than breast milk?		72.86%	88.95%	84.30%	19.48	1	0.000	0.20	86.89%	90.30%	86.36%	87.95%	1.30	2	0.522		0.05

## Data Availability

The data presented in this study are available on request from the corresponding author. The data are not publicly available due to privacy restrictions.
